# Experimental characterization of the thermo-optic coefficient vs. temperature for 4H-SiC and GaN semiconductors at the wavelength of 632 nm

**DOI:** 10.1038/s41598-023-37199-6

**Published:** 2023-06-23

**Authors:** Sandro Rao, Elisa D. Mallemace, Giuliana Faggio, Mario Iodice, Giacomo Messina, Francesco G. Della Corte

**Affiliations:** 1 Department DIIES, Mediterranea University, 89122 Reggio Calabria, Italy; 2 Institute of Applied Sciences and Intelligent Systems, Unit of Napoli. Napoli, 80131 Naples, Italy; 3 Department DIETI, University of Naples Federico II, 80125 Naples, Italy

**Keywords:** Electrical and electronic engineering, Electronics, photonics and device physics

## Abstract

The design of semiconductor-based photonic devices requires precise knowledge of the refractive index of the optical materials, a not constant parameter over the operating temperature range. However, the variation of the refractive index with the temperature, the thermo-optic coefficient, is itself temperature-dependent. A precise characterization of the thermo-optic coefficient in a wide temperature range is therefore essential for the design of nonlinear optical devices, active and passive integrated photonic devices and, more in general, for the semiconductor technology explored at different wavelengths, from the visible domain to the infrared or ultraviolet spectrum. In this paper, after an accurate ellipsometric and micro-Raman spectroscopy characterization, the temperature dependence of the thermo-optic coefficient ($\frac{\partial n}{\partial T}$) for 4H-SiC and GaN in a wide range of temperature between room temperature to T = 500 K in the visible range spectrum, at a wavelength of λ = 632.8 nm, is experimentally evaluated. For this purpose, using the samples as a Fabry–Perot cavity, an interferometric technique is employed. The experimental results, for both semiconductors, show a linear dependence with a high determination coefficient, R^2^ of 0.9648 and 0.958, for 4H-SiC and GaN, respectively, in the considered temperature range.

## Introduction

In the last years, wide band-gap semiconductors, such as Silicon Carbide (SiC) and Gallium Nitride (GaN), have gained interest in photonics due to their excellent optical and electronic properties, including the high thermal conductivity^1,2^, high refractive index^3,4^ and short lifetime for carriers^5,6^.

These properties make SiC and GaN promising candidates for various applications in photonics^7,8^.

To date, SiC has already been explored to make a variety of photonic devices, including light-emitting diodes (LEDs)^9,10^, photodiodes^11,12^ and, more important, for the design and fabrication of integrated photonic circuits thanks to the possibility of realizing low-loss waveguides^13^ leading to optically active devices, such as modulators^14,15^ or micro-ring resonators^16^. SiC has been shown to be compatible with Silicon (Si) processing technology, which means that it can be integrated with other Si-based photonic devices and circuits^17^. This could lead to the development of more compact and efficient photonic devices and circuits. In addition, SiC is also being studied for its nonlinear optical properties, which could make it useful for applications such as frequency conversion and optical signal processing^18,19^ also in high-power applications.

On the other hand, GaN is particularly interesting in the development of blue and green LEDs^20,21^, which are essential for energy-efficient lighting and displays, data storage and communications. In addition to its optical properties, GaN is also highly resistant to radiation and high temperatures^22^, making it suitable for use in harsh environments. Also in this case, several integrated photonic devices have been demonstrated^23,24^.

In all of said photonic applications, the knowledge of the precise value of the refractive index is of paramount importance for the correct design of devices, especially in those cases where resonance principles are utilized to carefully select the wavelength of operation, such as in ring resonators or multi-mode interference filters^16,19,25^. However, it is well known that temperature has a notable impact on refractive index, through a phenomenon known as the thermo-optic effect (TOE), which can negatively alter the device performance if it is not carefully taken into consideration in the design phase.

In our previous work, we measured both the thermo-optic coefficient (TOC) of a 4H-SiC and GaN at 1550 nm^26^, the most common wavelength used in optical communications due to the exceptionally low absorption losses shown by silica optical fibers, and the TOC dependence on temperature in the wide temperature range from RT to T = 480 K. However, both semiconductors are transparent in the shorter wavelength range of visible, which could favor the conception of new communication or sensory applications based on these materials, including biosensing^27^, nonlinear optics^28^, and quantum photonics^29^. For this reason, in this paper, we extend the data of^26^ with new measurements run at a wavelength close to 630 nm.

## Experimental method

**Figure 1:**
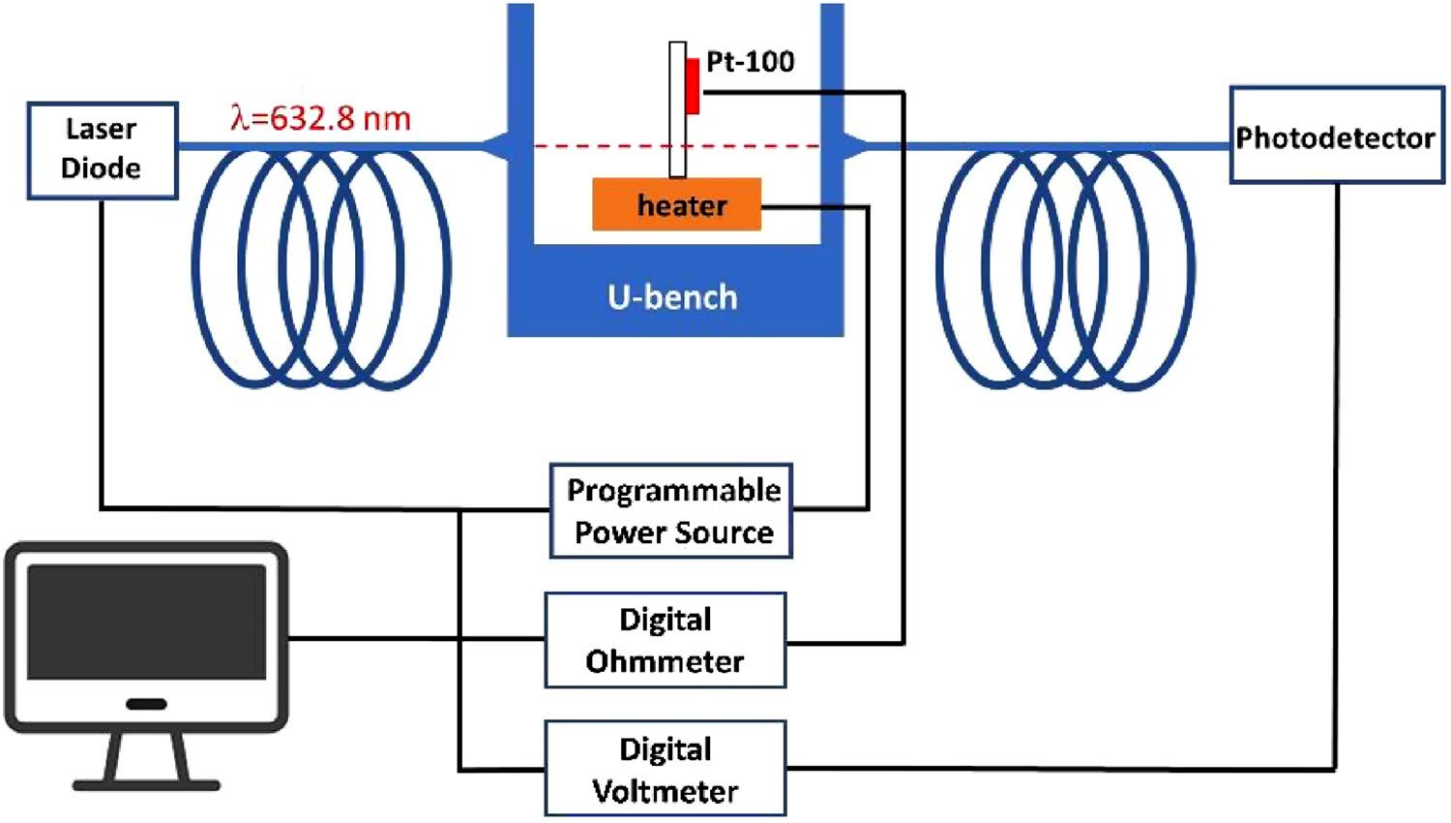
Schematic diagram of the experimental setup used for characterization of TOC as a function of temperature.

The thermo-optic coefficient of two <0001> oriented semi-insulating substrates of 4H-SiC and GaN^30^ were evaluated in a wide range of temperatures from room temperature (RT) to about T = 500 K at the wavelength of λ = 632.8 nm. The experimental setup, schematically illustrated in Fig. 1, is based on an interferometric technique. Since both the two thin samples are double-side polished at an optical grade, they behave as Fabry–Perot (FP) cavities. Specifically, a laser beam (Melles Griot, 05-LHP-927-S) at the wavelength of 632.8 nm is launched across the sample, orthogonally to the surface, and the transmitted signal is collected by a Si-photodetector^31^. The sample, contained in a U-bench (Thorlabs, FBC-1550-FC), is heated through a ceramic-resistive heater at a desired temperature, precisely monitored by a high-sensitive PT-100 sensor glued onto its surface. More information about the measurement technique and the experimental setup can be retrieved in^26^.

The transmitted signal *I*_*t*_ is the result of multiple interferences taking place inside the FP cavity, and it is given by the Airy formula:

1 $${I}_{t}=\frac{{I}_{0}}{1+\frac{4{F}^{2}}{{\pi }^{2}}{sin}^{2}\phi }$$

where *I*_*0*_ is the incident light intensity, $F=\pi \sqrt{R}/(1-R)$ is the reflecting finesse^32^ of the interferometric cavity, *R* is the reflectance of the two mirrors, and $\phi =2\pi nL/\lambda$ is the signal phase, with *λ* the wavelength of the incident light, and *n* and *L* the refractive index and the length of the cavity, respectively.

Since temperature affects both the refractive index (thermo-optic effect) and the FP cavity length (thermal expansion), the transmitted signal phase changes with temperature conferring a periodic shape. This behavior can be synthesized with the following formula^33^:

2 $$\frac{\partial \phi }{\partial T}=\frac{2\pi L}{\lambda }\left(\frac{\partial n}{\partial T}+\alpha \left(T\right)n(T)\right)$$

where *α(T)* is the thermal expansion coefficient, and $\frac{\partial n}{\partial T}$ is the thermo-optic coefficient. By measuring the pattern of the transmitted radiation intensity during the application of temperature ramps, it is possible to extract the refractive index variations with temperature, that is the thermo-optic coefficient.

The most important geometrical and physical parameters for 4H-SiC and GaN are reported in Table 1.

**Table 1:** Main geometrical and physical parameters.

Material	Substrate	Energy gap (eV) (T = 300 K)	Thickness L (mm)	Thermal expansion coefficient, α (10^–6^ K^−1^)	*n* (T = 300 K, λ = 632 nm)
4H-SiC	Semi-insulating <0001>	3.2	2	[34–36]	2.6526 (see Fig. 2)
GaN	Semi-insulating <0001>	3.43	0.35	[37, 38]	2.3780 (see Fig. 3)

It should be noted that the values reported in the literature for *α(T)* are rather spread both for 4H-SiC^34–36^ and GaN^37,38^; for this reason, the data contained in the respective references were all separately used to calculate $\frac{\partial n}{\partial T}.$

## Experimental results

### Ellipsometry

The samples used for measurements were obtained from BIOTAIN CRYSTAL^30^. Both of them are 5 × 5 mm^2^ dice cut from <0001> oriented substrates, with a thickness of 2 mm and 0.35 mm for 4H-SiC and GaN, respectively. The samples are semi-insulating, with resistivities of 10^5^ cm and 10^6^ cm, respectively.

**Figure 2:**
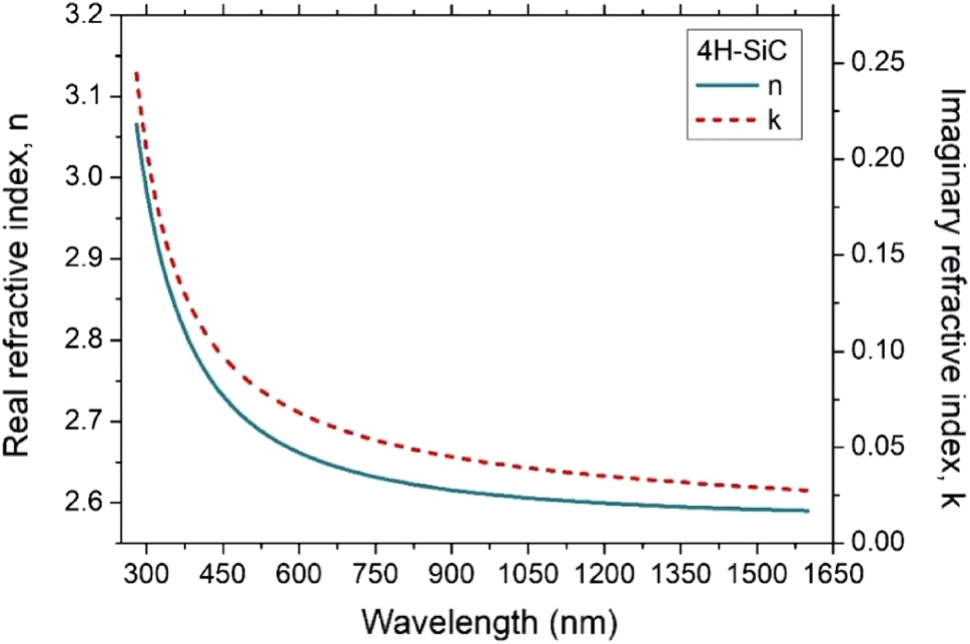
Real and imaginary refractive index of a semi-insulating 4H-SiC <0001> .

**Figure 3:**
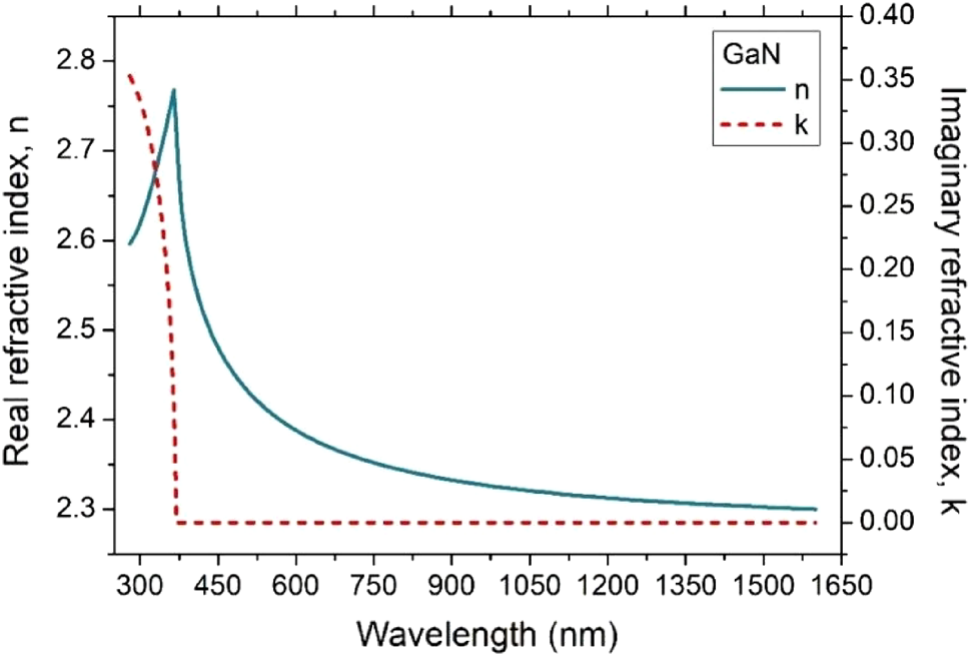
Real and imaginary refractive index of a semi-insulating GaN <0001> .

In order to evaluate the temperature dependence of the TOC, we started by measuring the real and imaginary refractive index of the two samples at room temperature. The optical properties were in particular characterized by a variable angle spectroscopic ellipsometer (UVISEL, Horiba, Jobin–Yvon) in a wide spectral range from ultraviolet to near-infrared, from λ = 280 to 1600 nm with a step of 5 nm. In Figs. 2 and 3, the real and imaginary refractive index for 4H-SiC and GaN are reported, respectively. The results achieved in this work are in good agreement with previously available literature results^39,40^.

### Micro-Raman spectroscopy

Raman spectroscopy, a fast and contactless measurement technique, was used to study crystalline quality and uniformity of the 4H-SiC and GaN wurtzite structure crystals^41,42^. As well known, Raman modes are representative of a unique crystal structure and it is therefore possible to obtain information on disorder, damage, lattice strain and impurities.

Raman spectra were collected by a HORIBA Scientific LabRAM HR Evolution Raman spectrometer with an integrated Olympus BX41 microscope using an 1800 lines/mm grating and a 100× objective. The spectral resolution of the Raman spectrometer was 0.2 cm^−1^.

**Figure 4:**
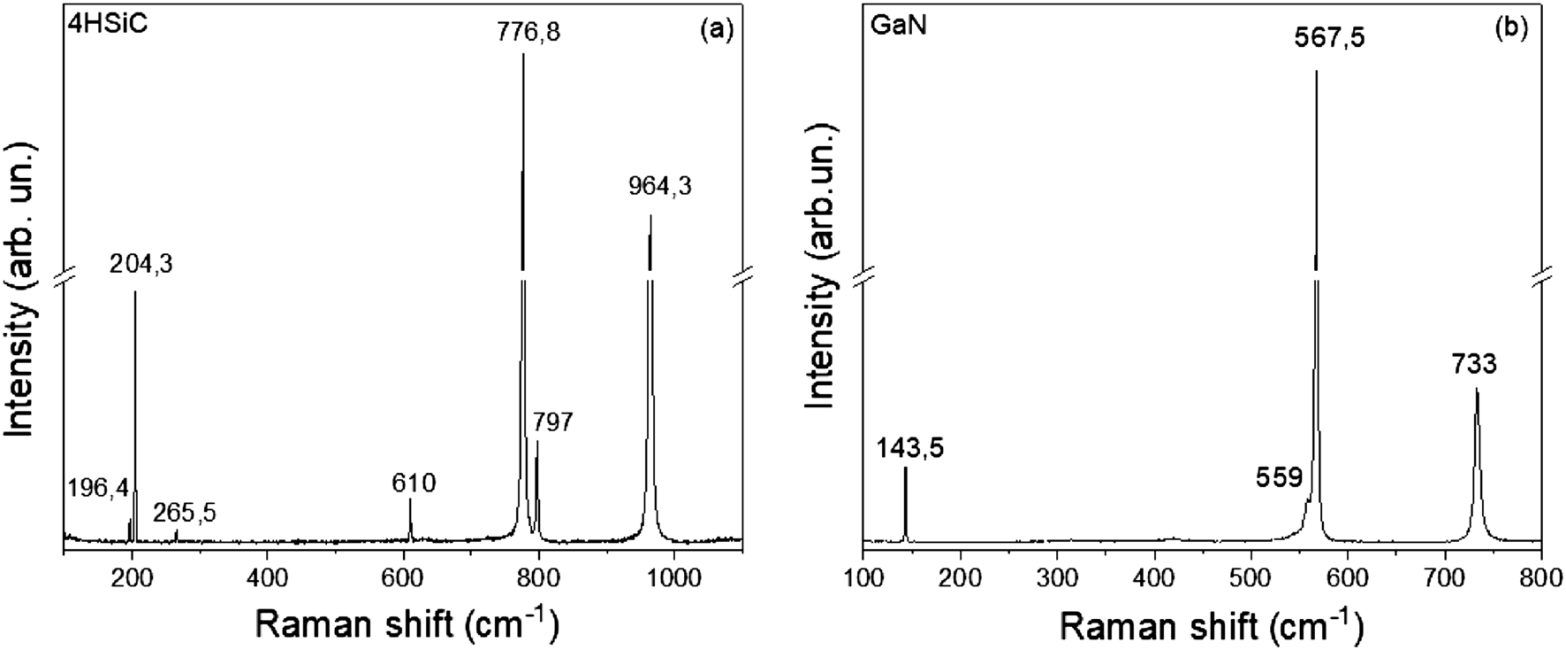
First order Raman spectra of 4H-SiC (**a**) and GaN (**b**).

Figure 4 shows the measured first-order Raman spectra from 4H-SiC (a) and GaN (b), taken at room temperature, in back scattering configuration with the incident laser beam (633 nm) normal to the sample surface (i.e. parallel to the c axis of the wurtzite structure crystals).

In the 4H-SiC Raman spectrum (Fig. 4a), the peak at 204.3 cm^−1^ is an E_2_ transverse acoustic (TA) mode, 610 cm^−1^ is an A_1_ longitudinal acoustic mode (LA), 776.8 cm^−1^ is an E_2_ transverse optical (TO) mode, 797 cm^−1^ is an E_1_ transverse optical (TO) mode and 964.3 cm^−1^ is an A_1_ longitudinal optical (LO) mode^41^. Furthermore, weaker peaks are observed in the spectrum at 196.4 and 265.5 cm^−1^ (E_2_ and E_1_ transverse acoustic mode, respectively).

Figure 4b shows the measured Raman spectrum from semi-insulating GaN. The predominant Raman peaks are at 143.5 and 568 cm^−1^ (E2 modes) and at 733 cm^−1^ (A1 longitudinal optical mode)^42^. The E1 (TO) phonon emerges at 559 cm^−1^ on the low-energy side of the E2 phonon.

The 4H-SiC and GaN Raman spectra measured are in accordance with the literature data^41–48^ and confirm the high crystalline quality of the two wide bandgap semiconductors.

### Thermo-optic coefficient

**Figure 5:**
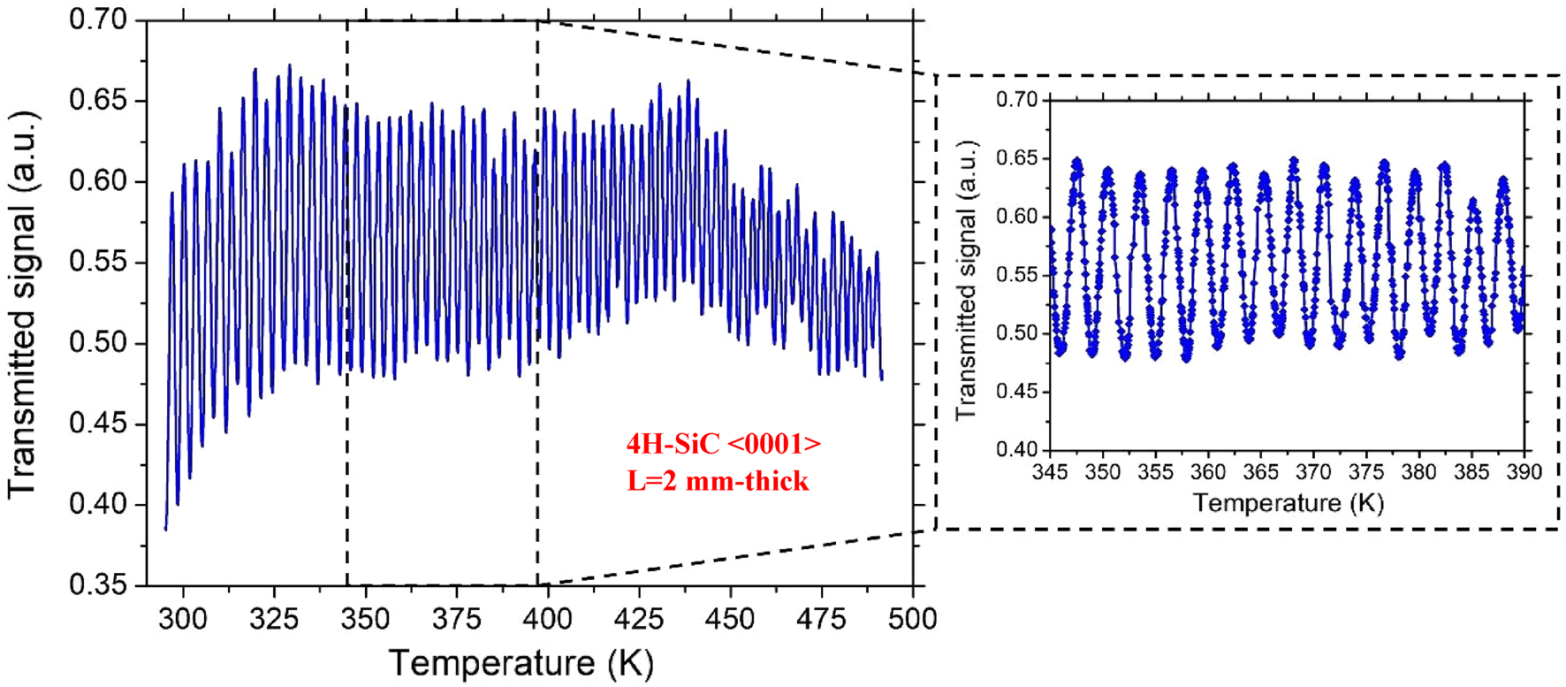
Transmitted signal as a function of temperature for the 4H-SiC sample.

**Figure 6:**
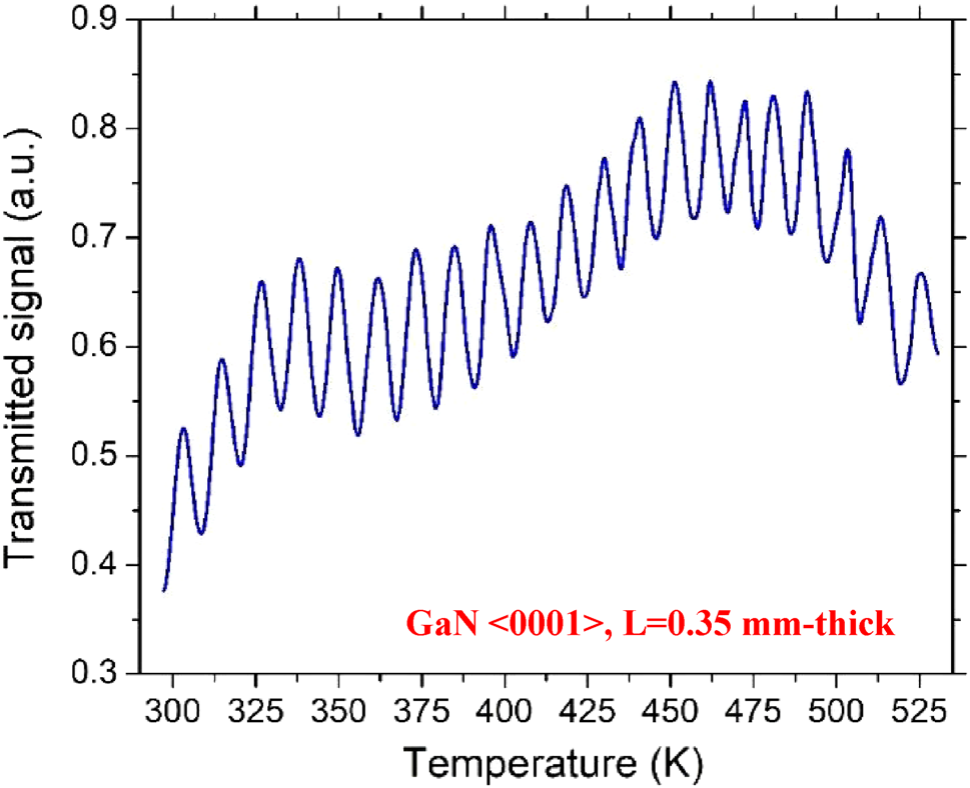
Transmitted signal as a function of temperature for the GaN sample.

To determine the thermo-optic coefficient, a continuous laser beam at the wavelength λ = 632.8 nm was launched on the FP cavity, and the transmitted signal was monitored and recorded while the temperature of the sample was slowly increased and monitored from RT to T = 500 K. The temperature dependence of the output transmitted signal for 4H-SiC and GaN samples are shown in Figs. 5 and 6, respectively.

It is worth noting that, due to the larger thickness of the 4H-SiC, the transmitted signal of this sample contained many more periods than that of GaN in the same temperature range.

In order to evaluate the TOC, the measurement of the distance, in terms of temperature, between two consecutive maxima (or minima) of the transmitted signal, corresponding in a phase shift of the optical propagation field of *Φ* = π, was estimated.

The evaluation of the TOC was started at room temperature using the refractive index, *n*(*T*), reported in Table 1. When the temperature increases, the TOC is evaluated by a recursive technique in which the thermal expansion coefficient, *α*(*T*), and refractive index *n*(*T*) are updated at each temperature step. Specifically, *α*(*T*) is calculated, for SiC and GaN, using several sets of data and relationships, as found in the references listed in Table 1, while *n*(*T*) is calculated according to (2) with the value extracted at the previous temperature step.

**Figure 7:**
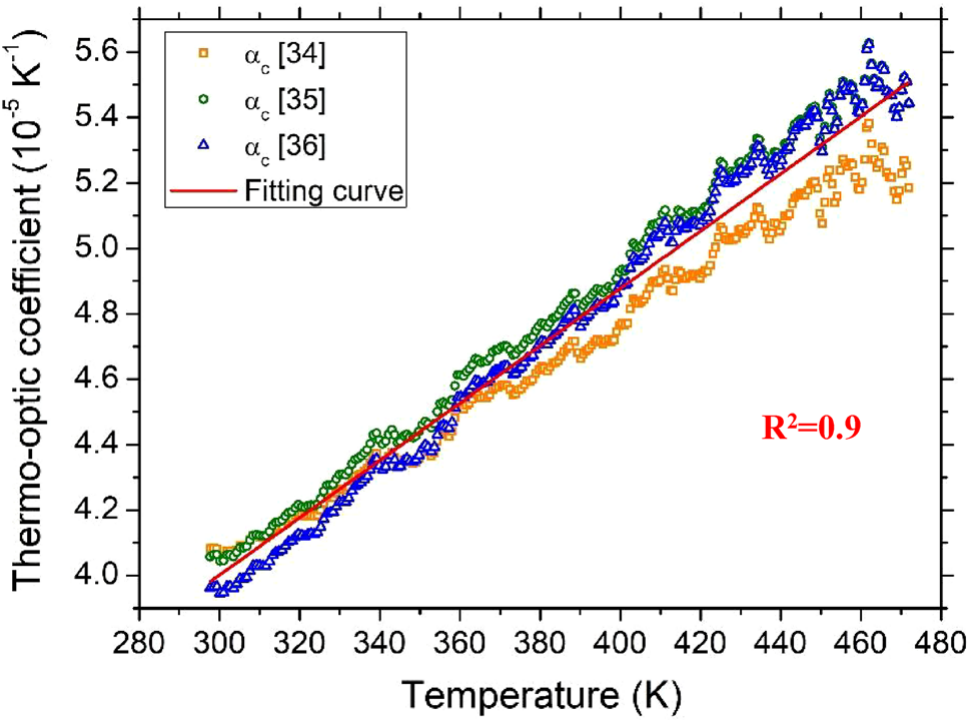
Thermo-optic coefficient as a function of temperature for 4H-SiC sample. The coefficient is separately calculated using the α(T) data reported in three references.

**Figure 8:**
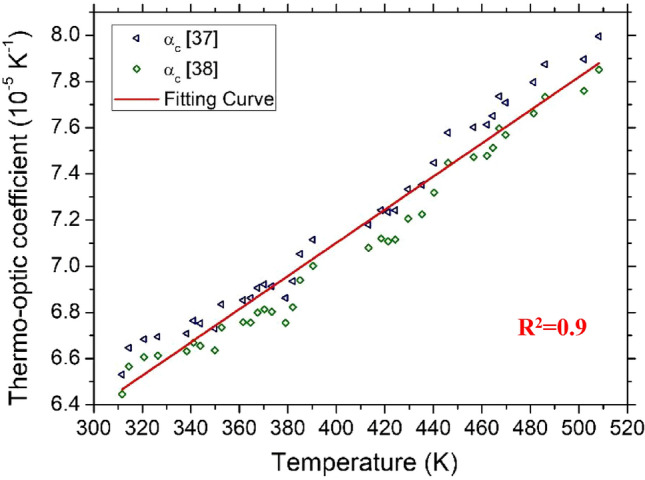
Thermo-optic coefficient as a function of temperature for GaN sample. The coefficient is separately calculated using the α(T) data reported in two references.

According to the above-detailed procedure, the δ*n*/δ*T* variations as a function of temperature for 4H-SiC and GaN are illustrated in Figs. 7 and 8, respectively.

The experimental data can be suitably described by a first-order-polynomial interpolation in the given temperature range, described by the following equations:

3 $$\frac{\partial n}{\partial T}=8.76\cdot {10}^{-8}T+1.37\cdot {10}^{-5} \mathrm{for } 4\mathrm{H}-\mathrm{SiC}$$

4 $$\frac{\partial n}{\partial T}=7.18\cdot {10}^{-8}T+4.23\cdot {10}^{-5}\mathrm{ for GaN}$$

In our analysis, the coefficient of determination, *R*^*2*^, was calculated in order to evaluate the agreement of experimental data $\frac{\partial n}{\partial T}$ vs* T* and the calculated best linear fit, *f*_*L*_*(T)*. Both samples show a high degree of linearity, with an *R*^2^ of 0.9648 and 0.9583 for 4H-SiC and GaN, as reported in Figs. 7 and 8, for 4H-SiC and GaN, respectively.

Another important parameter characterizing the goodness of the linear approximation of TOC vs. T is the root-mean-square error (*rmse*) of all of the experimental points and *f*_*L*_*(T).* We performed three cycles of measurements, with both positive and negative temperature ramps, in three different days, in order to evaluate the stability and minimize unavoidable measurement errors. The calculated *rmse* for 4H-SiC and GaN is 8.41 × 10^–7^ K^−1^ and 8.55 × 10^–7^ K^−1^, respectively.

Table 2 summarizes the calculated room temperature (RT) thermo-optic coefficients for 4H-SiC and GaN at 632.8 nm and 1550 nm, these latter taken from our previous work^26^. It can be observed that at RT, the TOC values at 632.8 nm are slightly higher than those measured at 1550 nm for both semicondutctors, but the measured TOC increase in GaN is greater than in 4H-SiC. A similar result was observed by Watanabe et al.^49^. In Fig. 4 of Ref.^49^, reporting the RT thermo-optic coefficients of 4H-SiC and GaN over an extended range of wavelengths, it can be seen that the variation of the TOC of 4H-SiC is gradual. On the contrary, $\frac{\partial n}{\partial T}.$ increases rapidly for GaN with decreasing wavelength. Such a different trend has been attributed by Watanabe et al. to the difference between the direct and indirect bandgaps of semiconductors.

**Table 2:** Room Temperature thermo-optic coefficient of 4H-SiC and GaN at 632.8 nm and 1550 nm (values from our previous work).

λ (nm)	4H-SiC	GaN
	TOC (10^–5^ K^−1^)	TOC (10^–5^ K^−1^)
632.8	4.10	6.60
1550	3.60	5.15

## Conclusions

In this work, the temperature dependence of the thermo-optic coefficient ($\frac{\partial n}{\partial T})$ of two wide bandgap semiconductors, i.e. 4H-SiC and GaN, at the wavelength λ = 632.8 nm is reported. First, the high crystalline quality and uniformity of the two sample was assessed by micro-Raman analysis. Thermal-optical measurements were performed in a wide range of temperatures from RT to T = 500 K using an interferometric method in a Fabry–Perot cavity with a length corresponding to the sample thickness while the material optical parameters were measured through ellipsometry at room temperature. The experimental data of $\frac{\partial n}{\partial T}$ as function of temperature has been well modeled with a linear function, with a high value of the determination coefficient, *R*^2^, of 0.9648 and 0.9583 for 4H-SiC and GaN, respectively, showing, moreover, good stability over three cycles of measurements.

To our knowledge, these are the first experimental results about TOC measurement and its dependence on temperature for both 4H-SiC and GaN, two semiconductors that will be largely explored for the design of a new generation of optoelectronic and photonic devices in the visible spectral range.

## Data Availability

The datasets used and/or analysed during the current study available from the corresponding author on reasonable request.
